# The Oncologic Value of Transoral Resection in Early-Stage Laryngeal Squamous Cell Carcinoma of the Glottis: A Retrospective Study

**DOI:** 10.7759/cureus.69975

**Published:** 2024-09-23

**Authors:** Athanasios Ioannidis, Georgios Louverdis, Aristeidis I Giotakis, Konstantinos Tarazis, Efthymios Kyrodimos

**Affiliations:** 1 First University Department of Otorhinolaryngology, Head and Neck Surgery, University of Athens, Hippocrateion Hospital, Athens, GRC; 2 Otolaryngology - Head and Neck Surgery, Ippokrateio General Hospital of Athens, Athens, GRC

**Keywords:** laryngeal neoplasms, laryngeal tumors, laser, prognosis, surgical managment

## Abstract

Background and objective

The treatment of early-stage laryngeal glottic cancer involves surgery or radiotherapy, and both have similar survival rates. However, discrepancies between systematic reviews in the literature point towards the continuous need for more data. In this study, we aimed to investigate the oncologic value of surgery at an ENT university department of a tertiary hospital in Greece.

Materials and methods

Patients with T_1_ or T_2_ laryngeal squamous cell carcinoma of the glottis who underwent transoral tumor resection between April 2014 and September 2021 at the hospital were deemed eligible for this study. Disease-free survival (DFS), local control rate (LCR), overall survival (OS), and laryngeal preservation were assessed.

Results

We enrolled 43 subjects with a median age of 67 years (range: 46-84 years). An overwhelming majority of the subjects were men (39/43). The most common stage was T_1a_ (22/43 subjects). OS was 74 months and DFS was 67 months. We noted local recurrence in 7/43 subjects. Three subjects passed away after five years of follow-up. Eventually, total laryngectomy was performed in 4/43 subjects, two of whom initially suffered from a T_2_ glottic carcinoma.

Conclusions

Our results align with the findings of the systematic reviews supporting high survival rates, laryngeal preservation, and avoidance of radiotherapy complications observed after transoral surgery for early-stage laryngeal glottic carcinoma.

## Introduction

The optimal treatment of early-stage laryngeal cancer, either T_1_ or T_2_ according to the VIII “Union for International Cancer Control” Edition, consists of surgery (nowadays mainly transoral resection) or radiotherapy [1-4]. These two treatment options have demonstrated similar oncological outcomes [5,6].

Several studies have compared the oncologic outcomes of transoral resection to radiotherapy. In their meta-analysis in 2007, Higgins et al. found no significant differences in local control rate (LCR) and overall survival (OS) between the two methods. However, there was a trend favoring OS after transoral resection [2]. In their meta-analysis in 2012, Abduherin et al. reported no significant differences in LCR and OS between surgery and radiotherapy for T_1a_ glottic tumors. However, they identified a higher organ preservation rate for patients initially treated with transoral laser resection [7]. A meta-analysis by Yoo et al. in 2014 reported no differences in LCR and OS, though five out of 10 included studies reported significantly higher OS after transoral resection [8]. Vaculik et al., in their meta-analysis in 2019, identified no differences in LCR between the two methods [9]. However, they reported significantly higher OS, disease-free survival (DFS), and organ preservation rates after transoral resection for T_1_ glottic tumors [9]. On the contrary, O’Hara et al. (2013) found similar LCR rates between the two treatments for T_1a_ tumors. However, they found slightly better LCR after radiotherapy for T_1b_ tumors [4].

Discrepancies between the systematic reviews and the ongoing debate about the optimal treatment of early-stage laryngeal cancer point towards the continuous need for more data. In light of this, we conducted this study intending to enhance the knowledge and awareness about this subject by retrospectively describing the oncologic outcomes of patients with early-stage laryngeal squamous cell carcinoma after transoral resection at the ENT university department of a tertiary hospital in Greece.

## Materials and methods

Study design and population

We retrospectively reviewed all patients treated with transoral resection for an early-stage laryngeal squamous cell carcinoma of the glottis (T_1a_, T_1b_, T_2_, Ν0, M0) at the institute between April 2014 and September 2021. This study was approved by the local institutional review board. The procedures followed adhered to the ethical standards of the Committee on Human Experimentation of the institution.

Data collection

The policy at our department favored transoral resection when deemed possible. Inclusion criteria involved patients fit for surgery, adequate exposure of the lesion, and capability of total resection of the tumor. Data were retrospectively collected and documented for each head and neck cancer patient. These data included age, gender, smoking and alcohol consumption, comorbidities, laryngeal subregion of the tumor and tumor TNM stage according to the VIII “Union for International Cancer Control” Edition, status of T (tumor size and site), of N (regional lymph node involvement) and M (distant metastasis), and grade and histology. Furthermore, the type of cordectomy according to the system proposed by the European Laryngological Society Working Committee was documented [10]. Further data included registration of recurrences and further treatment, LCR, DFS, and OS status along with the dates of the events, organ preservation rate, and cause of death. 

Surgical technique overview

All cases had undergone prior microlaryngoscopy for a preoperative biopsy. A pathologic diagnosis of squamous cell carcinoma was obtained. Staging was performed with CT scans or MRI scans of the neck with contrast and CT scans of the chest. Each treatment plan was approved by the multidisciplinary tumor board meeting.

The operation was performed under general anesthesia with an intubation tube of size 5.0 to 6.5; we inserted a plastic protection guard at the teeth of the upper jaw or a wet gauze at an upper jaw without teeth. Then, we exposed the tumor with a Zeitels operating laryngoscope (Endocraft, Warwick, RI) and we inspected the tumor with a surgical microscope. After the application of protective sheets around the laryngoscope, we set the power of the carbon dioxide (CO_2_) laser (Sharplan 20C CO_2_ laser; All States MED; Miami Lakes, FL) at 2-5 Watts on a continuous mode. We then resected the tumor along with a 1-2 mm healthy surrounding tissue. We applied traction with microforceps. Extra tissue at the resection border was excised for safety margin control. Neuro patties with adrenaline solution and/or a monopolar cautery were used for hemostasis.

Postoperatively, patients received intravenous antibiotics (mostly amoxicillin/clavulanic acid 1.2 grams three times daily) and inhaled a solution of corticosteroid (budesonide 1 mg thrice daily) with adrenalin three times daily on occasion. Nutrition, specifically a mechanical soft diet, was started in the afternoon soon after the surgery. Patients were discharged on the first postoperative day and received antibiotics orally (mostly amoxicillin/clavulanic acid a total dose of 2 grams divided into two doses daily).

Data analysis

Data were analyzed using the SPSS Statistics v26.0 (IBM Corp., Armonk, NY). Countable data were tabulated; for metric data, means, standard deviations (SD), and 95% confidence intervals (CI) were calculated. The Kaplan-Meier method was used to estimate survival rates. Survival curves were compared by using a log-rank test.

## Results

Clinicopathologic characteristics and treatment

A total of 43 subjects were included in the study. Data were made available by the head and neck surgeon of our department. The cohort's median age was 67 years (range 46-84 years) and 39 were men. Most subjects were heavy smokers. The most frequent tumor was T1a(22/43 subjects; Table 1). Most subjects underwent a type III cordectomy (24/43; Table 2). 

**Table 1:** Clinicopathological characteristics of the study subjects

Variable	Category	Frequency	%
Gender	Men	39	91%
	Women	4	9%
Smoking	Below 10 cigarettes daily	1	2%
	10-20 cigarettes daily	8	19%
	More than 20 cigarettes daily	34	79%
Alcohol consumption	None or occasionally	11	26%
	More than 1 glass of alcohol daily	32	74%
TNM staging	T_1a_	22	51%
	T_1b_	11	26%
	T_2_	10	23%
Histological grade	Low	13	30%
	Intermediate	20	47%
	High	10	23%

**Table 2:** Treatment and prognosis of the study subjects

Variable	Category	Frequency	%
Type of resection	II cordectomy	1	2%
	III cordectomy	24	56%
	IV cordectomy	8	18%
	Va cordectomy	5	12%
	Vb cordectomy	5	12%
Recurrence	Yes	7	16%
	No	36	84%
Treatment of recurrence	Total laryngectomy	4	9%
	Radiotherapy	2	5%
	Transoral laser resection	1	2%

Follow-up

Subjects were followed up for at least two years (mean follow-up time: 52 months, range: 24-120 months). Seven subjects developed local recurrence. Of them, one subject was initially staged as T_1a_, two as T_1b_, and four as T_2_. Four patients experienced recurrence within 15 months. Among the subjects with recurrence, four (two of which with an initial T_2_ glottic tumor) underwent total laryngectomy, two were treated with radiotherapy, and one underwent transoral laser revision resection (Table 2).

**Figure 1:**
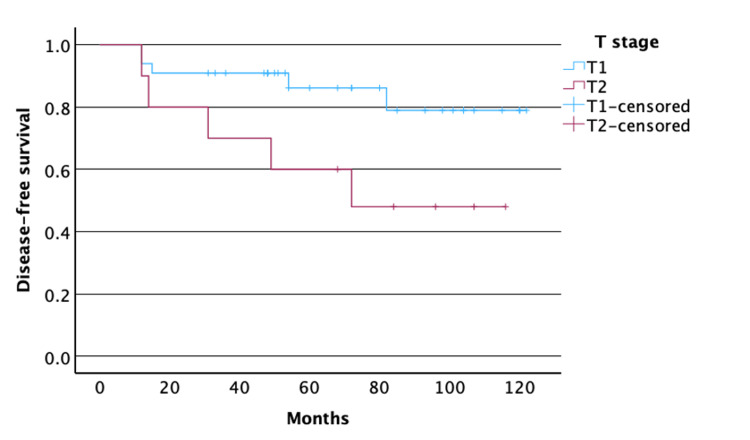
Kaplan-Meier survival curves for DFS in patients with T1 and T2 laryngeal glottic squamous cell carcinoma Y-axis: survival probability. X-axis: DFS in months. Patients with T_1_ laryngeal glottic squamous cell carcinoma had significantly higher DFS time compared to patients with T_2_ laryngeal glottic squamous cell carcinoma (p=0.043) DFS: disease-free survival

**Figure 2:**
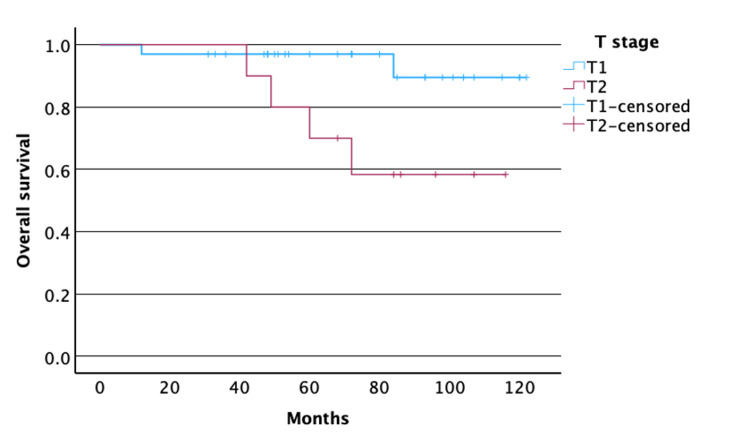
Kaplan-Meier survival curves for OS in patients with T1 and T2 laryngeal glottic squamous cell carcinoma Y-axis: survival probability. X-axis: OS in months. Patients with T_1_ laryngeal glottic squamous cell carcinoma had significantly higher OS time compared to patients with T_2_ laryngeal glottic squamous cell carcinoma (p=0.024) OS: overall survival

The two-year LCR was 96% (21/22) for T_1a_, 91% (10/11) for T_1b_, and 70% (7/10) for T_2_. Similarly, the five-year LCR was 96% (21/22), 82% (9/11), and 60% (6/10), respectively. Two and one more patients, initially staged as T_2_, had passed away by the two- and five-year follow-up, respectively. OS was 74 months and DFS was 67 months. We found that DFS and OS were better in the T_1_ group compared to the T_2_ group treated with transoral laser resection (p=0.043 and p=0.024, respectively; Figures 1-2).

## Discussion

A few discrepancies found among the systematic reviews that compare the benefits of surgery vs. radiotherapy for early-stage laryngeal squamous cell carcinoma of the glottis prompted us to investigate the oncologic value of surgery for this disease at our University Department of Otorhinolaryngology. Our results revealed two years without recurrence in over 90% of T_1a_ and T_1b_ tumors. Only one patient out of the 43 staged as T_1a_ had to undergo total laryngectomy as a salvage treatment one year after his initial treatment. This indicates the efficacy of transoral surgery in organ preservation. Only four patients in total had to undergo total laryngectomy after recurrence. Vaculik et al., Abduherin et al., and Yoo et al. have also reported the favorable organ preservation rates of transoral surgery over radiotherapy in their meta-analyses [4,7,9].

Our results align with the results of similar studies and revealed that transoral laser resection, especially for T_1a_ and T_1b_ laryngeal carcinomas, is a safe and effective treatment option [11-16]. Our local control rate for T_1a_ tumors was 96% (21/22), endorsing the efficacy of transoral resection as a first-line treatment for these patients. As per the recommendations of the National Institute for Health and Care Excellence proposed in 2016, we also offered transoral laser microsurgery for all patients with a newly diagnosed T_1a_ glottic carcinoma [17]. Many authors, however, were skeptical about transoral laser resection of T1b tumors, especially when those invaded the anterior commissure [4,18]. In our study, local control of T_1b_ was 91% (10/11) and 82% (9/11) after two and five years of follow-up, respectively. Other studies have reported similar outcomes [19]. In our study, all T_1b_ patients have survived until now, indicating a very promising survival outcome for this stage.

Studies reporting radiotherapy in patients with T_1b_ glottic laryngeal squamous cell carcinoma have shown a similar five-year LCR to our results, ranging between 83 and 94% [1,20] Sval et al. revealed a pattern for opting primary radiotherapy for T_1b_ glottic cancers in the SEER registry; our results revealed that these patients could benefit from a transoral laser resection, with similar oncological outcomes and without the complications of radiotherapy [21]. Indeed, most recurrences were observed in T_2_ tumors (4/7), raising the issue of whether radiotherapy should be reserved for these cases. Other studies have also reported reduced LCR in T_2_ of around 75%, specifically for tumors with impaired vocal cord mobility or when anterior commissure is involved [5,17,22]. The variation in our result could be due to our small sample as well as to a probable higher anterior commissure involvement, which was not documented. Kaplan-Meier survival curve revealed that T_2_ tumors had lower DFS and OS than T_1_ tumors (Figures 1-2).

Studies investigating radiotherapy as a treatment for T_2_ laryngeal cancer have shown a wide range of local control rates, from 48 to 97.6% [22]. The vertical involvement of the anterior commissure and the impaired mobility of the vocal cords have been implicated as a significant factor in worse outcomes, and several studies have recommended a combined chemoradiotherapy approach for such cases [20,23-25]. A monotherapy approach, i.e., involving only radiotherapy or surgery, might not be always feasible for these patients. Some studies have proposed subcategorization of T_2_ cases with such characteristics and, subsequently, different treatment [1,20,22].

Our study has some limitations, such as its retrospective design. The most significant limitation was the lack of comparison of transoral surgery results with the outcomes in subjects undergoing radiotherapy for early-stage laryngeal squamous cell carcinoma. Furthermore, our sample size was relatively small. However, our cohort comprised only those patients treated by the head and neck surgeon in our department. On an annual basis, it would amount to six patients with early-stage glottic squamous cell carcinoma undergoing transoral surgery by a single surgeon. This is a relatively large number, given that many patients favor radiotherapy instead of surgery, and twice as many patients present with late-stage instead of early-stage laryngeal squamous cell carcinoma in Greece [26,27]. Occasionally, patients with early-stage laryngeal squamous cell carcinoma were treated by other surgeons as well. However, these patients were not included in the study due to limited collected data and/or because these surgeons were no longer members of our department at the time of the study.

Another limitation of this study is the inadequate collection of data on anterior commissure involvement. Furthermore, it would be interesting to use patient-reported measures to document the speech and swallowing function. The Therapy Outcome Measure of Speech and Language could aid in the proper documentation of postoperative voice and swallowing function.

## Conclusions

Currently, there are no established criteria favoring either transoral resection or radiotherapy in the treatment of early-stage laryngeal glottic cancer, and the choice in most cases depends on the clinician’s judgment and the patient’s preference. Based on our findings, transoral laser resection of early-stage laryngeal squamous cell carcinoma of the glottis is associated with high survival rates, preservation of the laryngeal organ, and avoidance of radiotherapy complications. Our study highlights that transoral laser resection is highly beneficial and can lead to excellent outcomes in T_1a_-and T_1b_-staged laryngeal carcinomas.
